# Facilitating smoking cessation in patients who smoke: a large-scale cross-sectional comparison of fourteen groups of healthcare providers

**DOI:** 10.1186/s12913-019-4527-x

**Published:** 2019-10-25

**Authors:** E. Meijer, R. M. J. J. Van der Kleij, N. H. Chavannes

**Affiliations:** Public Health and Primary Care, Leiden University Medical Center, Hippocratespad 21, PO Box 9600, 2300 RC Leiden, The Netherlands

**Keywords:** Smoking cessation care, Tobacco dependence guideline, Ask-advise-refer model, Implementation, Barriers, Role identity, Physicians, Regression tree analyses

## Abstract

**Background:**

Although healthcare providers are well placed to help smokers quit, implementation of smoking cessation care is still suboptimal. The Ask-Advise-Refer tasks are important aspects of smoking cessation care. We examined to which extent a large and diverse sample of healthcare providers expressed the intention to implement smoking cessation care and which barriers they encountered. We moreover examined to which extent the Ask-Advise-Refer tasks were implemented as intended, and which determinants (in interaction) influenced intentions and the implementation of Ask-Advise-Refer.

**Methods:**

Cross-sectional survey among addiction specialists, anaesthesiologists, cardiologists, general practitioners, internists, neurologists, paediatricians, pulmonologists, ophthalmologists, surgeons, youth specialists, dental hygienists, dentists, and midwives (*N =* 883). Data were analysed using multivariate linear and logistic regression analyses and regression tree analyses.

**Results:**

The Ask-Advice-Refer tasks were best implemented among general practitioners, pulmonologists, midwives, and addiction specialists. Overall we found a large discrepancy between asking patients about smoking status and advising smokers to quit. Participants mentioned lack of time, lack of training, lack of motivation to quit in patients, and smoking being a sensitive subject as barriers to smoking cessation care. Regression analyses showed that the most important determinants of intentions and implementation of Ask-Advise-Refer were profession, role identity, skills, guideline familiarity and collaboration agreements for smoking cessation care with primary care. Determinants interacted in explaining outcomes.

**Conclusions:**

There is much to be gained in smoking cessation care, given that implementation of Ask-Advise-Refer is still relatively low. In order to improve smoking cessation care, changes are needed at the level of the healthcare provider (i.e., facilitate role identity and skills) and the organization (i.e., facilitate collaboration agreements and guideline familiarity). Change efforts should be directed towards the specific barriers encountered by healthcare providers, the contexts that they work in, and the patients that they work with.

## Background

The negative health consequences of smoking tobacco are widely known. However, a considerable number of people continue to smoke [1]. Healthcare providers (HCPs) are well placed, and -according to clinical guidelines and the World Health Organization- have the responsibility to discourage the use of tobacco and counsel smokers in their quit attempts [2–4]. Many smokers are motivated to quit smoking for health reasons, and a large number of Dutch ex-smokers stated that their quit attempt had been motivated by a HCP’s advice to quit smoking [5, 6]. HCPs have different types of effective smoking cessation interventions at their disposal, including very brief advice, nicotine replacers and pharmacotherapy, behavioural counselling, and e-health interventions [7–11]. Clinical guidelines provide HCPs with an overview of these interventions and describe how smoking cessation care (SCC) should be provided [3, 12].

For different types of HCPs, different opportunities exist to facilitate smoking cessation. Certain HCPs such as general practitioners (GPs), dentists and dental hygienists mostly provide care to ‘healthy’ smokers, who are otherwise not seen by HCPs, and the majority of smokers visits their GP or dentist at least annually [2, 13, 14]. However, many smokers report that they were not advised to quit smoking when visiting their GP or dentist [2, 6, 15]. Other HCPs, including medical specialists, see smokers who suffer (an even higher risk of developing) smoking-related conditions, making smoking cessation care even more important. Although smoking-related complaints in patients may facilitate the provision of SCC [16], this is not necessarily the case [17, 18]. For example, a recent multinational study showed that primary care physicians and pulmonologists who were frustrated by chronic obstructive pulmonary disease (COPD) patients’ smoking behaviours were less inclined to provide SCC [17].

Given that smoking has many different negative health consequences, the provision of SCC is relevant to many disciplines within healthcare. The downside of this might be that few HCPs perceive smoking cessation care specifically as their responsibility or as part of their role [19, 20]. Indeed, role identity appears relatively low among many types of HCPs [18, 21, 22], but role identity is not sufficient to explain implementation failure or success. The consolidated framework for implementation research (CFIR) states that the implementation of interventions depends on factors related to the intervention itself, the ‘inner and outer setting’ in which the intervention resides, the HCP (which includes role identity), and the implementation process [23]. In line with this framework, research into determinants of implementation of clinical guidelines for SCC shows that aspects such as wording and format are important for implementation success [24–27]. With regard to the inner and outer setting, lack of time, reimbursement and referral possibilities, and environments unsupportive of SCC have been found to hamper implementation [16, 28–33]. HCPs may furthermore be less likely to provide SCC to patients without smoking-related complaints, when they perceive that patients are unmotivated to quit or do not want professional help, or that SCC harms the relationship with patients [16, 29, 34, 35]. Finally, with regard to HCP factors, HCP’s outcome expectancies, attitude, self-efficacy, level of training, knowledge or skills, and own smoking history are important, among other factors [16, 17, 29, 31, 32, 35–44].

Studies comparing the implementation of SCC for different groups of HCPs revealed striking contrasts in levels of implementation as well as barriers to SCC [22, 31, 44, 45]. It therefore does not seem desirable or practical to ask all HCPs to implement every element of SCC. Instead, more limited models of SCC such as the Ask-Advise-Refer and Ask-Advise-Connect models are more appropriate [46]. These models aim for collaboration between HCPs, suggesting that all HCPs ask about smoking status and advise to quit, and then refer to SCC specialists for further counselling. Recent studies among GPs, pulmonologists, surgeons and anaesthesiologists in the United States suggest that this approach is feasible [33, 44].

The current study investigated among a large sample of HCPs for whom SCC is relevant:

- Their intentions to implement SCC as described in the Dutch Tobacco Dependence Guideline, which determinants were associated with their intentions to implement SCC and whether determinants interacted;
- Which barriers they named towards the implementation of SCC in general as described in the guideline;
- If they implemented part of SCC, namely the Ask-Advise-Refer tasks, as intended (dosage delivered); which determinants were associated with dosage delivered of Ask-Advise-Refer and whether determinants interacted.

## Method

### Design

Observational cross-sectional study. The STROBE guidelines were used for reporting [47].

### Participants and procedure

Data were collected in The Netherlands between February and November 2017, using an online survey (some addiction specialists filled out a hardcopy version of the survey at a conference). Participants were eligible if they were practicing physicians, dental hygienists, dentists or midwives. The study was introduced as a questionnaire about their opinion on SCC, their experiences with SCC, and the barriers and facilitators that they encountered. In order to prevent selection bias, we explicitly stated that participants could take part regardless of experience in SCC, and we employed a wide range of recruitment strategies (e.g., through professional associations who sent out an invitation to participate to their members, participants who forwarded the study invitation to their colleagues (snowball sampling), e-mails sent directly to relevant departments of all hospitals in The Netherlands). Participants were recruited primarily through their professional associations (45%) or colleagues (24%), see Additional file 1: Table S1 for details. One thousand two hundred twenty-two people started with the survey, of whom 883 completed it and were included in this study (72%). The final sample included 45 addiction specialists, 62 anaesthesiologists, 52 cardiologists, 148 GPs, 63 internists, 63 neurologists, 36 paediatricians, 102 pulmonologists, 16 ophthalmologists, 68 surgeons, 48 youth specialists, 31 other physicians, 38 dental hygienists, 26 dentists, and 65 midwives. As such, participants were a mixture of HCPs working within and outside of hospitals. Of these, 25 participants had not yet completed medical specialist training.

Participants were informed that participation was voluntarily and that data would be analysed and stored anonymously and treated confidentially. They provided informed consent before filling out the survey. Median time needed to complete the questionnaire was 13 min. Four gift coupons of € 100.- and 10 of € 50.- were distributed among participants who completed the survey. The procedure was cleared for ethics by the Medical Ethical Committee of Leiden University Medical Center.

### Measures

Multiple variables were measured, of which those relevant to this study are described below (more detail can be found elsewhere [48]. The selection and operationalization of variables was based on previous work on determinants of implementation of SCC [37, 39, 49–55]. Unless indicated otherwise, variables did not have missing values.

#### Predictor variables

##### Participant and patient characteristics

Participants provided their gender, year of birth (2 missing), profession, number of years worked as professional (1 missing), previous participation in SCC training, and smoking status (never smoker/ex-smoker/current smoker).

##### Guideline familiarity and presence

Participants indicated *familiarity* with the previous versions and revised version of the guideline; GPs answered questions about the guideline with regard to the general practice smoking cessation guideline produced by Dutch College of General Practitioners. Answer categories were [1] ‘I do not know it’, [2] ‘I have heard about it, but not read it’, [3] ‘I browsed through it’, [4] ‘I have largely familiarized myself with it’, [5] ‘I have completely familiarized myself with it’ (3–5 were recoded into ‘Read’ for the analyses). Participants also indicated whether previous versions of the guideline were *present* at their place of work, recoded into ‘yes’ (hardcopy, digital, or both) and ‘no’ (absent, or do not know), 8 missing.

##### Determinants of implementation

Answer categories for psychosocial characteristics were [1] ‘completely disagree’ – [5] ‘completely agree’, with [6] ‘do not know/inapplicable’ (recoded into [3] ‘agree nor disagree’), unless indicated otherwise. We measured, with one item each, *agreement* with the guideline’s content (‘I agree with the content of the guideline’), *attitude* (‘I find it important that the guideline is implemented correctly’), *knowledge* and *skills* (1 missing) (‘I have sufficient knowledge/skills to implement the guideline correctly’, respectively), *social support* (‘I feel supported in implementing the guideline’), *role identity* (‘As a [profession], I see it as my role to implement the guideline correctly’), and *outcome expectations* (‘If I use the guideline correctly, more patients will successfully quit smoking’), see Additional file 1: Table S2 for means/standard deviations on psychosocial variables per HCP group.

Participants indicated to what extent 14 pre-specified factors were *barriers* to guideline implementation and providing SCC, with answer categories [1] ‘not at all’, [2] ‘not’, [3] ‘slightly’, [4] ‘strongly’, and [5] ‘very strongly’. Barriers assessed were lack of guideline adaptability (‘The guideline cannot be adapted to the context that I work in’), guideline complexity (‘The guideline is too complex to use’), task interference (‘Other things I have to do get in the way’), lack of time (‘I have insufficient time’), lack of materials (‘There are insufficient materials available’), lack of patient reimbursement (‘SCC is insufficiently reimbursed’), lack of referral possibilities (‘There are insufficient referral possibilities for patients that want to quit’), professional’s reward (‘I receive insufficient rewards for implementing the guideline’), lack of training (‘I have had insufficient training in SCC), sensitive subject (‘(Quitting) smoking is a sensitive subject for patients’), patient’s resistance (‘Patients are negative about SCC’), patients’ lack of motivation (‘Patients are unmotivated to quit’), patients’ dishonesty (‘Patients are dishonest about their smoking behaviour; 1 missing), and the impact on patient-provider relationship (‘Implementing the guideline negatively affects my relationship with the patient’). Participants could also indicate other barriers that they encountered.

Participants indicated whether they themselves, or their department/organization had arranged *collaboration agreements* for SCC with *primary care* (e.g., GPs, psychologists, SCC coaches) and *secondary care* (7 missing), with answer categories ‘no’, ‘yes’, and ‘do not know’ (recoded into ‘no’). Finally, participants indicated whether SCC was financed (through regular budget, sponsors, healthcare insurance companies, or other means), or not.

#### Outcome variables

##### Barriers to implementation

See Predictor variables.

##### Intention to use the guideline

Participants rated their agreement with ‘I intend to implement the guideline correctly’, [1] ‘completely disagree’ – [5] ‘completely agree’, with [6] ‘do not know/inapplicable’ (recoded into [3] ‘agree nor disagree’).

##### Implementation of ask-advise-refer

Participants indicated, via self-report, the dosage delivered of the tasks ‘Ask’ about smoking status (of all patients); ‘Advise’ to quit smoking, in a clear and personalized way (of patients who smoke) and ‘Refer’ to adequate SCC (of patients motivated to quit). Answer categories were [1] ‘all’, [2] ‘the majority’, [3] ‘half’, [4] ‘the minority’, and [5] ‘none’. For the analyses, we dichotomized Ask (all vs. majority-none), and Advise and Refer (all/majority vs. half-none), based on the median.

### Statistical analyses

Analyses were performed on data from participants with full data on all variables that were included in the analyses (see Measures). Attrition analyses were performed using t-tests and χ^2^-tests. We first performed univariate linear regression analyses for intentions to use the guideline. Predictors that were significantly associated with intention were included in the multivariate linear regression model. We then performed a set of regression tree analyses [56] with intentions as the outcome, using all predictors that were used in the linear regression analyses. This procedure examines in a data-driven manner whether predictor variables interact, and searches for optimal cut-off values in predictor variables. The minimum number of participants per leaf was fixed at 10, and the minimum increase in fit (complexity parameter) was set at 0.0001. For the remaining parameters we used default options. The selection process of the initial, non-pruned tree was performed 1000 times. Regression tree analyses were performed using the Rpart package version 4.1–9 in R statistical software version 3.2.5 [57, 58]. Effect size was calculated by constructing new categorical variables that represented the terminal nodes, which were used in a one-way ANOVA (resulting in ƞ_p_^2^). Frequencies were calculated for barriers to implementation and dosage delivered of Ask-Advise-Refer. We then examined determinants of dosage delivered of the Ask-Advise-Refer tasks through univariate logistic regression analyses. These were followed by three multivariate logistic regression models for the respective outcomes, using predictors that were significant in the univariate analyses. For interactions between determinants of Ask-Advise-Refer, we performed three sets of regression tree analyses. Correct classification rates (CCRs) based on the final regression tree models were calculated for dosage delivered variables, which were compared to a priori CCRs (i.e., all participants assigned to the largest category). Only regression trees with more than one split were presented visually.

## Results

### Preliminary analyses

Attrition analyses showed that completion of the survey was unrelated to age, number of years worked, and dosage delivered of ask, advise and refer. Participants who completed the survey had stronger intentions to use the guideline, were more often male and less often dental hygienists, youth specialists, or ‘other’ physicians (see Additional file 1: Table S3 for descriptive statistics and attrition analyses).

### Intentions to implement the guideline

Descriptive statistics for outcome variables are shown in Table 1. Intentions to use the guideline appeared strongest among midwives (Table 1) and were quite similar among the other HCP groups.

**Table 1:** Intentions to use the guideline, and dosage delivered of Ask, Advise and Refer by profession (*N* = 883)

	*N*	Intention *M (SD)*	Ask	Advise	Refer
Physicians					
Addiction specialist	45	3.98 (0.81)	83%	56%	40%
Anaesthesiologist	62	3.18 (0.98)	77%	18%	15%
Cardiologist	52	3.25 (0.65)	73%	62%	50%
GP	148	3.70 (0.81)	11%	51%	64%
Internist	63	3.21 (0.74)	84%	46%	44%
Neurologist	63	3.21 (0.81)	52%	27%	41%
Paediatrician	36	3.67 (0.76)	22%	28%	53%
Pulmonologist	102	3.50 (0.79)	88%	56%	84%
Other	31	3.42 (0.99)	45%	32%	36%
Ophthalmologist	16	3.19 (0.54)	0%	25%	25%
Surgeon	68	3.24 (0.76)	54%	38%	49%
Youth specialist	48	3.38 (0.84)	19%	19%	21%
Other healthcare professionals					
Dental hygienist	58	3.45 (0.82)	64%	41%	26%
Dentist	26	3.50 (0.71)	62%	12%	35%
Midwife	65	4.25 (0.77)	99%	65%	68%

‘Intention’ ranges from 1 to 5, with higher scores indicating stronger intentions. ‘Ask’ concerns all patients; ‘Advise’ concerns all or the majority of smokers; ‘Refer’ concerns all or the majority of smokers motivated to quit. *GP* general practitioner

The multivariate linear regression model showed that GPs had stronger intentions to use the guideline than cardiologists, internists, and pulmonologists (see Table 2). Furthermore, intentions were stronger among participants with shorter work experience, more positive attitudes, and stronger skills, perceived social support and role identity. In addition, participants who agreed with the content of the guideline, and were familiar with previous versions had stronger intentions to use the guideline.

**Table 2:** Explaining intentions to use the guideline: linear regression analyses, *N* = 867

Predictor variables	Univariate	Multivariate	
	*b* (95% confidence interval)	*b* (95% confidence interval)	β
Participant characteristics			
Age	−0.01 (− 0.01;0.00)**	0.01 (− 0.01;0.01)	0.06
Gender (male)	− 0.28 (− 0.39;-0.16)***	− 0.07 (− 0.17;0.03)	− 0.04
Profession			
GP (ref.)	0	0	
Addiction specialist	0.28 (0.01;0.55)*	0.02 (− 0.25;0.30)	0.01
Anaesthesiologist	−0.52 (− 0.75;-0.28)***	− 0.22 (− 0.46;0.03)	−0.07
Cardiologist	−0.45 (− 0.70;-.19)**	−0.29 (− 0.53;-0.05)*	−0.08*
Internist	−0.49 (− 0.73;-0.35)***	−0.26 (− 0.49;-0.02)*	−0.08*
Neurologist	−0.49 (− 0.73;-0.25)***	−0.17 (− 0.42;0.07)	−0.05
Paediatrician	−0.03 (− 0.32;0.26)	− 0.05 (− 0.32;0.22)	−0.01
Pulmonologist	− 0.20 (− 0.40;0.01)	−0.21 (− 0.39;-0.02)*	−0.08*
Other	−0.36 (− 0.62;-0.9)	−0.09 (− 0.34;0.16)	−0.02
Surgeon	−0.46 (− 0.69;-0.23)***	−0.12 (− 0.35;0.12)	−0.04
Youth specialist	−0.32 (− 0.58;-0.06)*	−0.23 (− 0.48;0.02)	−0.06
Dental hygienist	−0.25 (− 0.49; 0.00)*	−0.09 (− 0.34;0.17)	0.03
Dentist	−0.20 (− 0.53;0.14)	0.14 (− 0.17;0.45)	0.03
Midwife	0.55 (0.32;0.78)***	0.24 (0.00;0.49)	0.07
Years worked	−0.01 (− 0.01; 0.00)*	−0.01 (− 0.02;0.00)*	−0.15*
SCC training	0.39 (0.27;0.52)***	−0.06 (− 0.18;0.07)	−0.03
Smoking status			
Never (ref.)	0		
Ex-smoker	−0.14 (−0.27;-0.01)*	− 0.05 (− 0.16;0.05)	−0.03
Current	−0.25 (− 0.51; 0.01)	−0.12 (− 0.33;0.09)	−0.03
Psychosocial factors			
Attitude	0.55 (0.49; 0.60)***	0.33 (0.26;0.39)***	0.33***
Knowledge	0.21 (0.16; 0.26)***	0.03 (−0.03;0.08)	0.03
Skills	0.25 (0.19; 0.30)***	0.08 (0.03;0.14)**	0.09**
Social support	0.29 (0.23; 0.34)***	0.09 (0.04;0.15)**	0.10**
Role identity	0.42 (0.38;0.47)***	0.16 (0.11;0.22)***	0.20***
Outcome expectations	0.37 (0.30; 0.44)***	0.04 (−0.02;0.15)	0.04
Lack of training^a^	−0.08 (− 0.13;-0.04)**	0.05 (0.00;0.09)	0.06
Guideline factors			
Agreement content	0.54 (0.45;0.62)***	0.15 (0.06;0.24)**	0.11**
Guideline presence	0.21 (0.09;0.33)**	−0.10 (−0.24;0.04)	−0.06
Guideline familiarity			
Unfamiliar (ref.)	0	0	
Heard of	0.31 (0.18;0.44)***	0.17 (0.06;0.28)**	0.09**
Read	0.59 (0.46;0.73)***	0.19 (0.03;0.35)*	0.10*
Lack of guideline adaptability^a^	−0.15 (−0.20;-0.08)***	0.02 (−0.04;0.09)	0.02
Guideline complexity^a^	−0.20 (− 0.27;-0.13)***	−0.03 (− 0.10;0.04)	−0.03
Environmental factors			
Collaboration primary care	0.15 (0.03;0.28)*	0.01 (−0.10;0.12)	0.00
Collaboration secondary care	0.24 (0.08;0.40)**	0.05 (−0.09;0.17)	0.02
Financial budget	0.03 (−0.10;0.16)		
Lack of patient reimbursement^a^	0.02 (−0.03;0.08)		
Lack of professional rewards^a^	−0.04 (− 0.09;0.01)		
Lack of time^a^	−0.08 (− 0.13; − 0.02)**	0.00 (− 0.06;0.05)	0.00
Task interference^a^	−0.10 (− 0.16;-0.05)***	−0.03 (− 0.09;0.03)	−0.04
Lack of materials^a^	−0.07 (− 0.13;-0.01)*	0.03 (− 0.02;0.08)	0.04
Lack of referral possibilities^a^	−0.04 (− 0.10;0.02)		
Patient barriers			
Smoking sensitive subject^a^	0.02 (−0.03;0.07)		
Negative towards smoking cessation care^a^	−0.02 (− 0.08;0.04)		
Unmotivated to quit^a^	−0.03 (− 0.09;0.03)		
Dishonest about smoking^a^	0.00 (−0.06; 0.06)		
Impact patient-provider relationship^a^	−0.02 (− 0.09;0.05)		

Multivariate model *R*^2^ = 0.48, Model *F* (37,829) = 20.55, *p* < .001

*GP* general practitioner, ‘Other’ profession includes ophthalmologists, *SCC* smoking cessation care; ^a^barriers to guideline implementation and provision of smoking cessation care

*p < .05, ** *p* < .01, *** *p* < .001

**Fig. 1:**
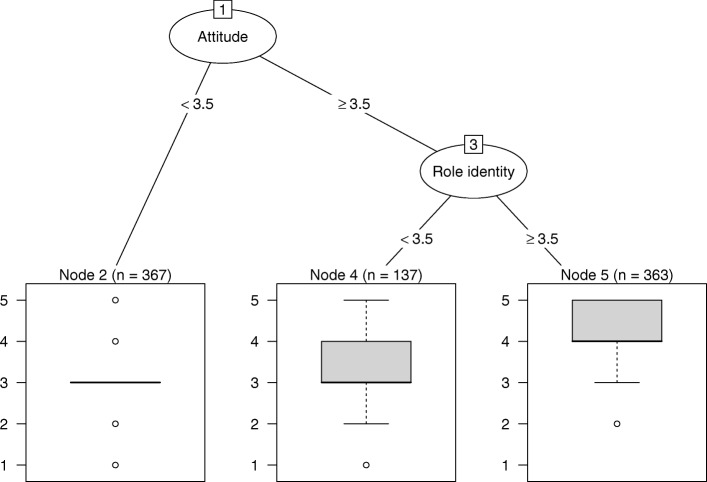
Regression tree explaining intentions to use the guideline

Regression tree analysis showed that intentions to use the guideline were explained by attitude and role identity (see Fig. 1), ƞ_p_^2^ = 0.32. Participants with less positive attitudes toward the guideline had relatively low intentions to implement the guideline (attitude < 3.5; mean intention 3.01). Role identity was important for participants with positive attitudes, such that those who perceived implementing the guideline as their task had stronger intentions (role identity ≥3.5, mean intention 4.05) than those with weaker role identities (role identity < 3.5, mean intention 3.33).

### Barriers to implementation of SCC as described in the guideline

The main barriers in the entire sample were lack of time, lack of training, lack of motivation to quit in patients, and smoking being a sensitive subject to discuss with patients (see Table 3). Pulmonologists reported the lowest level of barriers, and addiction specialists and paediatricians reported only lack of patient reimbursement and lack of training as strong barriers in 50% of these subsamples, respectively. Among anaesthesiologists, on the other hand, five barriers were reported by at least 50% of the subsample, which were lack of training, lack of time, task interference, smoking as a sensitive subject, and unmotivated patients.

**Table 3:** Barriers to guideline usage and provision of smoking cessation care by profession (*N* = 883)

			Guideline		Environment						Patient				
	N	SCC training professional	Adaptability	Complexity	Patient reimbursement	Professional rewards	Time	Task interference	Materials	Referral possibilities	Sensitive subject	Negative SCC	Unmotivated	Dishonest	Relationship
Physicians															
Addiction specialist	45	18%	7%	4%	**58%**	16%	24%	27%	16%	18%	22%	4%	27%	5%	2%
Anaesthesiologist	62	**61%**	13%	8%	24%	18%	**57%**	**52%**	34%	19%	**52%**	36%	**65%**	39%	8%
Cardiologist	52	48%	8%	4%	27%	19%	**50%**	44%	25%	29%	33%	14%	29%	27%	2%
GP	148	24%	4%	5%	39%	33%	45%	35%	8%	17%	28%	16%	31%	12%	3%
Internist	63	**54%**	18%	6%	37%	11%	38%	44%	22%	29%	33%	22%	**56%**	8%	0%
Neurologist	63	**64%**	10%	6%	19%	14%	**61%**	49%	29%	24%	35%	21%	35%	8%	0%
Paediatrician	36	**53%**	8%	6%	14%	8%	44%	31%	14%	6%	42%	19%	39%	19%	8%
Pulmonologist	102	35%	8%	5%	45%	25%	44%	34%	15%	24%	43%	23%	40%	20%	0%
Other	31	**55%**	10%	10%	10%	13%	39%	42%	36%	13%	23%	26%	42%	3%	0%
Ophthalmologist	16	**63%**	25%	0%	19%	0%	**56%**	**50%**	25%	6%	38%	38%	44%	38%	0%
Surgeon	68	**53%**	4%	7%	24%	22%	**56%**	41%	16%	22%	**52%**	37%	47%	37%	7%
Youth specialist	48	**65%**	25%	8%	19%	15%	**65%**	**65%**	31%	8%	48%	27%	**52%**	23%	10%
Other healthcare professionals															
Dental hygienist	58	**59%**	7%	7%	24%	28%	40%	26%	29%	21%	**60%**	33%	36%	29%	14%
Dentist	26	**58%**	15%	12%	15%	27%	46%	39%	23%	19%	**50%**	39%	54%	35%	27%
Midwife	65	26%	0%	5%	39%	37%	**54%**	40%	20%	17%	**57%**	31%	49%	35%	20%
Total	883	45%	10%	6%	31%	22%	48%	40%	21%	19%	41%	24%	42%	21%	6%

Percentages refer to participants who indicated that a factor was a ‘strong’ or ‘very strong’ barrier. Percentages ≥50 are indicated in bold

*GP* general practitioner, *SCC* smoking cessation counselling

In addition, in response to the open-ended question, 514 participants mentioned factors that complicated their implementation of the guideline. Some of the factors that were already assessed were repeated here. In addition, many participants mentioned that they were unfamiliar with the guideline. This was most common among dentists (50% of those who answered the open-ended question) and paediatricians (45%), and least common among GPs (3%) and addiction specialists (6%). Some participants reported perceptions of smoking that likely are unhelpful (e.g., habit, coping strategy), or patients characteristics that complicated SCC (e.g., lower intelligence, serious comorbidities, limited life expectancy). Organizational factors included difficulty to obtain pharmacotherapy in time, administrative burden of arranging patient reimbursement, low priority for SCC in their organization or among colleagues, and colleagues being smokers. Finally, several participants stated that the government should play a larger role in decreasing smoking prevalence.

### Implementation of the ask-advise-refer tasks

Midwives and pulmonologists most frequently reported that they asked their patients about smoking status, midwives and cardiologists most frequently advised smokers to quit, and pulmonologists most frequently referred motivated smokers to SCC (see Table 1). Examination of other SCC tasks showed that addiction specialists, GPs, midwives, and pulmonologists most frequently assisted smokers in their quit attempt. Furthermore, most participants indicated that they advised most smokers with smoking-related complaints and most pregnant smokers to quit smoking (see Additional file 1: Table S4), such that quit advice was provided more often to specific groups of patients than to smokers in general**.**

The multivariate logistic regression model for Asking about smoking status showed that GPs were significantly less likely than the other HCPs to ask all of their patients about smoking status - with the exception of paediatricians, with whom no significant differences were found (see Table 4). Furthermore, participants who had heard of, or read, the guideline were more likely to ask about smoking status than those who were unfamiliar with it.

**Table 4:** Explaining dosage delivered of Ask (all patients): Logistic regression analyses, *N* = 867

Predictor variables	Odds ratio (95% confidence interval)	
	Univariate	Multivariate
Participant characteristics		
Age	0.98 (0.97;0.99)**	0.99 (0.95;1.03)
Gender (male)	0.77 (0.59;1.01)	
Profession		
GP (ref.)	1	1
Addiction specialist	38.16 (15.15;96.10)***	56.31 (16.33;194.14)***
Anaesthesiologist	28.29 (12.84;62.31)***	50.14 (17.83;141.05)***
Cardiologist	22.39 (10.03;49.98)***	30.70 (11.18;84.32)***
Internist	43.73 (18.65;102.50)***	59.24 (20.61;170.31)***
Neurologist	9.08 (4.43;18.58)***	13.29 (4.90;36.08)***
Paediatrician	2.36 (0.92;6.04)	2.95 (0.98;8.89)
Pulmonologist	61.88 (27.94;137.02)***	66.00 (26.47;164.57)***
Other	3.50 (1.55;7.89)**	5.17 (1.89;14.18)**
Surgeon	9.85 (4.89;19.93)***	14.89 (5.68;39.01)***
Youth specialist	1.90 (0.78;4.64)	2.91 (1.01;8.39)*
Dental hygienist	14.54 (6.90;30.64)***	22.80 (8.01;64.91)***
Dentist	13.20 (5.13;33.97)***	21.98 (6.64;72.74)***
Midwife	528.00 (68.50;4069.68)***	647.02 (79.21;5735.64)***
Years worked	0.98 (0.97;0.99)**	1.01 (0.97;1.05)
SCC training	1.14 (0.85;1.54)	
Smoking status		
Never (ref.)	1	
Ex-smoker	0.92 (0.68;1.24)	
Current	0.64 (0.34;1.18)	
Psychosocial factors		
Attitude	1.14 (0.97;1.33)	
Intention	1.12 (0.96;1.31)	
Knowledge	1.07 (0.95;1.20)	
Skills	1.09 (0.95;1.26)	
Social support	0.91 (0.79;1.05)	
Role identity	1.13 (0.99;1.29)	
Outcome expectations	0.95 (0.80;1.13)	
Lack of training^a^	1.000 (0.89;1.13)	
Guideline factors		
Agreement content	0.72 (0.57;0.89)**	0.93 (0.66;1.31)
Guideline presence	0.40 (0.29;0.53)***	0.69 (0.38;1.24)
Guideline familiarity		
Unfamiliar (ref.)	1	1
Heard of	1.48 (1.07;2.04)*	1.69 (1.11;2.58)*
Read	0.80 (0.58;1.11)	3.01 (1.52;5.99)**
Lack of guideline adaptability^a^	0.96 (0.83;1.12)	
Guideline complexity^a^	0.90 (0.76;1.06)	
Environmental factors		
Collaboration primary care	1.13 (0.83;1.54)	
Collaboration secondary care	2.49 (1.64;3.77)***	1.40 (0.80;2.43)
Financial budget	0.65 (0.48;0.88)**	1.22 (0.80;1.86)
Lack of patient reimbursement^a^	1.15 (1.01;1.31)*	1.03 (0.84;1.25)
Lack of professional rewards^a^	0.98 (0.87;1.10)	
Lack of time^a^	0.94 (0.83;1.07)	
Task interference^a^	1.01 (0.89;1.14)	
Lack of materials^a^	1.16 (1.01;1.32)*	1.02 (0.85;1.23)
Lack of referral possibilities^a^	1.16 (1.01;1.33)*	1.15 (0.93;1.43)
Patient barriers		
Smoking sensitive subject^a^	1.05 (0.94;1.18)	
Negative towards smoking cessation care^a^	0.98 (0.85;1.13)	
Unmotivated to quit^a^	0.99 (0.86;1.14)	
Dishonest about smoking^a^	0.91 (0.79;1.05)	
Impact patient-provider relationship^a^	0.92 (0.78;1.08)	

*GP* general practitioner, ‘Other’ profession includes ophthalmologists, *SCC* smoking cessation care; ^a^barriers to guideline implementation and provision of smoking cessation care

Multivariate model Cox & Snell *R*^2^ = 0.36, Nagelkerke *R*^2^ = 048, Model χ^2^(24) = 392.93, *p* < .001

* *p* < .05, ** *p* < .01, *** *p* < .001

The multivariate model for Advising patients to quit showed that participants were more likely to advise all, or the majority of their patients who smoked to quit if they reported stronger skills and role identity, and had collaboration agreements on SCC with primary care (see Table 5). In addition, participants who believed that following the guideline would negatively impact their relationship with the patient, were less likely to advise to quit.

**Table 5:** Explaining dosage delivered of advise (all or the majority of smokers): Logistic regression analyses, *N* = 868

Predictor variables	Odds ratio (95% confidence interval)	
	Univariate	Multivariate
Participant characteristics		
Age	1.01 (1.00;1.02)	
Gender (male)	1.00 (0.76;1.31)	
Profession		
GP (ref.)	1	1
Addiction specialist	1.18 (0.61;2.32)	0.71 (0.27;1.82)
Anaesthesiologist	0.20 (0.10;0.42)***	0.93 (0.36;2.41)
Cardiologist	1.52 (0.80;2.89)	4.26 (1.82;9.97)**
Internist	0.81 (0.45;1.46)	2.30 (1.01;5.24)*
Neurologist	0.35 (0.18;0.67)**	1.19 (0.49;2.91)
Paediatrician	0.36 (0.16;0.81)*	0.82 (0.30;2.22)
Pulmonologist	1.20 (0.72;1.99)	1.58 (0.83;3.02)
Other	0.40 (0.20;0.81)*	1.07 (0.44;2.63)
Surgeon	0.59 (0.33;1.05)	2.00 (0.87;4.61)
Youth specialist	0.22 (0.10;0.48)***	0.63 (0.24;1.67)
Dental hygienist	0.67 (0.36;1.24)	1.83 (0.78;4.28)
Dentist	0.12 (0.04;0.43)**	0.36 (0.09;1.46)
Midwife	1.73 (0.95;3.16)	3.19 (1.32;7.68)*
Years worked	1.01 (1.00;1.03)	
SCC training	2.58 (1.91;3.50)***	1.35 (0.87;2.09)
Smoking status		
Never (ref.)	1	
Ex-smoker	1.24 (0.92;1.68)	
Current	0.94 (0.50;1.76)	
Psychosocial factors		
Attitude	1.13 (1.05;1.44)*	0.85 (0.66;1.09)
Intention	1.62 (1.37;1.92)***	1.27 (0.98;1.65)
Knowledge	1.56 (1.37;1.77)***	1.01 (0.83;1.21)
Skills	1.98 (1.68;2.33)***	1.39 (1.12;1.72)**
Social support	1.45 (1.24;1.69)***	1.08 (0.89;1.31)
Role identity	1.74 (1.50;2.01)***	1.43 (1.16;1.77)**
Outcome expectations	1.24 (1.04;1.47)*	0.87 (0.68;1.11)
Lack of training^a^	0.67 (0.59;0.76)***	0.92 (0.77;1.10)
Guideline factors		
Agreement content	1.71 (1.37;2.14)***	1.06 (0.76;1.49)
Guideline presence	1.99 (1.48;2.66)***	1.11 (0.67;1.83)
Guideline familiarity		
Unfamiliar (ref.)	1	1
Heard of	1.44 (1.03;2.00)*	0.98 (0.65;1.47)
Read	3.38 (2.41;4.74)***	1.47 (0.83;2.61)
Lack of guideline adaptability^a^	0.85 (0.73;0.99)*	0.96 (0.78;1.18)
Guideline complexity^a^	0.85 (0.71;1.00)	
Environmental factors		
Collaboration primary care	2.39 (1.76;3.26)***	1.50 (1.03;2.19)*
Collaboration secondary care	2.73 (1.86;4.01)***	1.53 (0.97;2.21)
Financial budget	1.29 (0.95;1.76)	
Lack of patient reimbursement^a^	1.31 (1.15;1.48)***	
Lack of professional rewards^a^	1.08 (0.96;1.21)	
Lack of time^a^	0.82 (0.72;0.93)**	0.99 (0.81;1.21)
Task interference^a^	0.76 (0.67;0.86)***	0.93 (0.75;1.15)
Lack of materials^a^	0.96 (0.84;1.10)	
Lack of referral possibilities^a^	0.99 (0.87;1.14)	
Patient barriers		
Smoking sensitive subject^a^	0.88 (0.79;1.00)*	1.04 (0.87;1.24)
Negative towards smoking cessation care^a^	0.84 (0.73;0.96)*	1.15 (0.92;1.45)
Unmotivated to quit^a^	0.70 (0.60;0.81)***	0.81 (0.65;1.00)
Dishonest about smoking^a^	0.77 (0.67;0.90)**	0.96 (077.;1.18)
Impact patient-provider relationship^a^	0.63 (0.53;0.75)***	0.69 (0.55;0.88)**

*GP* general practitioner; ‘Other’ profession includes ophthalmologists, *SCC* smoking cessation care; ^a^barriers to guideline implementation and provision of smoking cessation care

Multivariate model Cox & Snell *R*^2^ = 0.22, Nagelkerke *R*^2^ = 0.30, Model χ^2^(36) = 215.87, *p* < .001

* *p* < .05, ** *p* < .01, *** *p* < .001

The multivariate model for Referring patients showed that GPs were significantly more likely to refer smokers than addiction specialists, anaesthesiologists, and youth specialists, but less likely than pulmonologists (see Table 6). Furthermore, participants were more likely to refer smokers if they were male, had participated in SCC training, perceived social support for using the guideline, and were familiar with the guideline. In addition, presence of patient reimbursement and collaboration agreements for SCC with primary care were associated with more referrals.

**Table 6:** Explaining dosage delivered of Refer (all or the majority of smokers motivated to quit): Logistic regression analyses, *N* = 868

Predictor variables	Odds ratio (95% confidence interval)	
	Univariate	Multivariate
Participant characteristics		
Age	1.00 (0.99;1.02)	
Gender (male)	0.71 (0.54;0.93)*	0.50 (0.35;0.71)***
Profession		
GP (ref.)	1	1
Addiction specialist	0.37 (0.19;0.74)**	0.23 (0.09;0.61)**
Anaesthesiologist	0.10 (0.04;0.21)***	0.24 (0.09;0.65)**
Cardiologist	0.56 (0.29;1.06)	1.08 (0.47;2.48)
Internist	0.45 (0.25;0.81)**	0.94 (0.42;2.14)
Neurologist	0.39 (0.21;0.72)**	0.86 (0.37;2.01)
Paediatrician	0.62 (0.30;1.30)	0.86 (0.37;2.02)
Pulmonologist	3.00 (1.60;5.63)**	4.35 (2.04;9.25)***
Other	0.26 (0.13;0.53)***	0.49 (0.20;1.19)
Surgeon	0.53 (0.29;0.94)*	1.52 (0.67;3.44)
Youth specialist	0.15 (0.07;0.32)***	0.22 (0.09;0.57)**
Dental hygienist	0.20 (0.10;0.28)***	0.41 (0.17;1.01)
Dentist	0.30 (0.12;0.71)**	0.88 (0.30;2.61)
Midwife	1.17 (0.63;2.17)	1.08 (0.44;2.63)
Years worked	1.00 (0.99;1.01)	
SCC training	2.15 (1.59;2.92)***	1.61 (1.02;2.56)*
Smoking status		
Never (ref.)	1	
Ex-smoker	0.99 (0.73;1.33)	
Current	0.60 (0.32;1.13)	
Psychosocial factors		
Attitude	1.23 (1.05;1.43)*	1.09 (0.86;1.39)
Intention	1.38 (1.18;1.62)***	1.07 (0.84;1.38)
Knowledge	1.29 (1.14;1.46)***	1.03 (0.85;1.24)
Skills	1.21 (1.05;1.39)*	0.90 (0.73;1.11)
Social support	1.44 (1.24;1.67)***	1.35 (1.11;1.65)**
Role identity	1.32 (1.15;1.50)***	1.00 (0.82;1.21)
Outcome expectations	1.12 (0.95;1.33)	
Lack of training^a^	0.80 (0.71;0.90)***	1.06 (0.88;1.26)
Guideline factors		
Agreement content	1.37 (1.10;1.71)**	0.89 (0.63;1.25)
Guideline presence	2.43 (1.81;3.28)***	1.29 (0.78;2.14)
Guideline familiarity		
Unfamiliar (ref.)	1	1
Heard of	2.47 (1.78;3.42)***	1.81 (1.22;2.69)**
Read	3.44 (2.46;4.82)***	1.38 (0.77;2.50)
Lack of guideline adaptability^a^	0.79 (0.67;0.92)**	1.00 (0.80;1.27)
Guideline complexity^a^	0.83 (0.70;0.98)*	1.00 (0.77;1.30
Environmental factors		
Collaboration primary care	3.23 (2.34;4.46)***	1.92 (1.31;2.83)**
Collaboration secondary care	2.86 (1.91;4.27)***	1.35 (0.83;2.18)
Financial budget	1.94 (1.42;2.64)***	1.86 (1.26;2.76)**
Lack of patient reimbursement^a^	0.99 (0.88;1.12)	
Lack of professional rewards^a^	1.00 (0.90;1.12)	
Lack of time^a^	0.94 (0.83;1.07)	
Task interference^a^	0.84 (0.74;0.95)**	
Lack of materials^a^	0.86 (0.75;0.98)*	1.12 (0.93;1.34)
Lack of referral possibilities^a^	0.85 (0.74;0.97)*	0.85 (0.71;1.02)
Patient barriers		
Smoking sensitive subject^a^	0.96 (0.85;1.08)	
Negative towards smoking cessation care^a^	0.94 (0.81;1.07)	
Unmotivated to quit^a^	0.86 (0.75;0.99)*	0.96 (0.80;1.16)
Dishonest about smoking^a^	0.94 (0.81;1.09)	
Impact patient-provider relationship^a^	0.84 (0.72;0.99)*	0.91 (0.74;1.13)

*GP* general practitioner; ‘Other’ profession includes ophthalmologists, *SCC* smoking cessation care; ^a^barriers to guideline implementation and provision of smoking cessation care

Multivariate model Cox & Snell *R*^2^ = 0.24, Nagelkerke *R*^2^ = 0.32, Model χ^2^(35) = 234.45, *p* < .001

* *p* < .05, ** *p* < .01, *** *p* < .001

Finally, we examined whether determinants interacted in explaining implementation of the Ask-Advise-Refer tasks. The regression tree analysis for Ask resulted in a tree with one split on profession, CCR = 78% (a priori CCR = 57%). Specifically, GPs, paediatricians, youth specialists, and ‘other physicians’ were less likely to ask all patients about smoking status (probability ask 0.17) than other groups (probability ask 0.75).

**Fig. 2:**
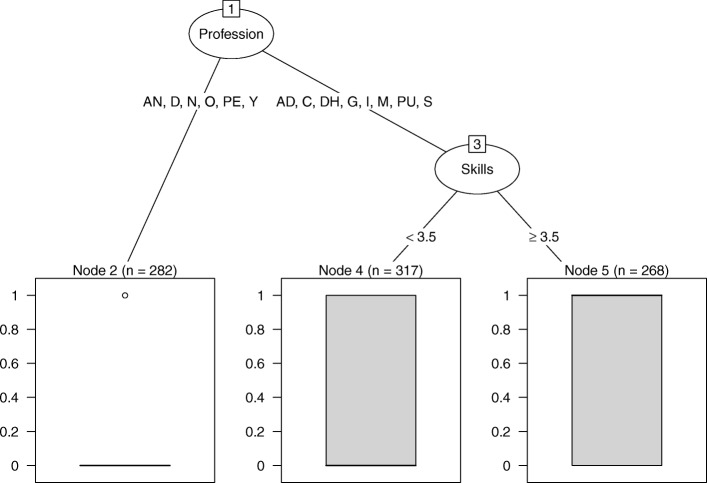
Regression tree explaining advising smokers to quit smoking. *Note*. AD = addiction specialist, AN = anaesthesiologist, C = cardiologist, D = dentist, DH = dental hygienist, G = general practitioner, I = internist, M = midwife, N = neurologist, O = other physician, PE = paediatrician, PU = pulmonologist, S = surgeon, Y = youth specialist

Regression tree analysis for Advise showed that profession and skills interacted in explaining provision of quit advice (see Fig. 2), CCR = 67% (a priori CCR = 58%). Results showed that anaesthesiologists, dentists, neurologists, ‘other physicians’, paediatricians and youth specialists were unlikely to advise ‘all or the majority’ of smokers to quit (probability advise 0.23). Among the other groups, those with lower skills were less likely to provide quit advice (skills < 3.5; probability advise 0.40) than those with higher skills (skills ≥3.5; probability advise 0.66).

**Fig. 3:**
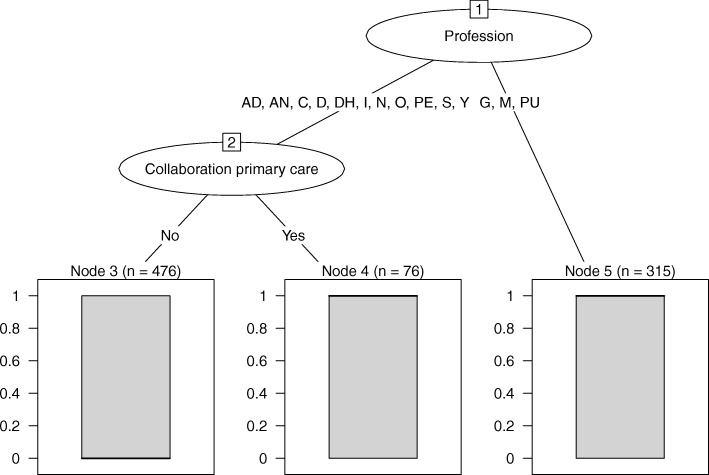
Regression tree explaining referring smokers for smoking cessation care. *Note*. AD = addiction specialist, AN = anaesthesiologist, C = cardiologist, D = dentist, DH = dental hygienist, G = general practitioner, I = internist, M = midwife, N = neurologist, O = other physician, PE = paediatrician, PU = pulmonologist, S = surgeon, Y = youth specialist

For Refer, the regression tree showed that profession and collaboration agreements for SCC with primary care interacted in explaining whether participants referred patients for SCC (see Fig. 3), CCR = 68% (a priori CCR = 51%). GPs, midwives and pulmonologists were quite likely to refer ‘all or the majority’ of smokers motivated to quit (probability refer 0.71). Among the other groups, the small group of participants who reported collaboration agreements were far more likely to refer patients (probability refer 0.64) than the large group of participants without such agreements (probability refer 0.32).

## Discussion

Among 14 groups of HCPs, we examined the intention to implement SCC and which determinants influenced this intention. Moreover, we assessed what barriers HCPs experienced towards the implementation of SCC. Finally, we examined if the Ask-Advice-Refer tasks were implemented as intended, and which determinants influenced the implementation of the Ask-Advise-Refer tasks. This study extended previous work by including and comparing 14 groups of HCPs. It was the first to examine whether determinants interact in explaining intentions to implement SCC and implementation of smoking cessation (i.e., the Ask-Advise-Refer tasks).

Intention to implement SCC was quite similar among HCPs, except for the midwives who indicated a stronger intention to implement. The Ask-Advise-Refer tasks were best implemented by GPs, pulmonologists, midwives, and addiction specialists, although for all HCPs there remains room for improvement. Across groups a large discrepancy was found between asking patients about smoking status and advising smokers to quit, which may communicate implicit approval of smoking. Anaesthesiologists in particular asked about smoking status relatively often, but typically refrained from providing quit advice, confirming previous studies [59, 60].

The most important barriers to implement SCC were lack of time, lack of training, perceived lack of motivation to quit in patients, and smoking being a sensitive subject to discuss with patients. In terms of the CFIR [23], the factors represent a mixture of inner and outer setting factors, and HCP factors. Although these barriers have been identified before [16, 17, 30–32, 36], they were not significantly associated with intentions and implementation of Ask-Advise-Refer in the regression models. Results instead showed that the most important determinants of intentions and implementation were profession, role identity, skills, guideline familiarity and collaboration agreements for SCC with primary care. As such, the CFIR domain HCP factors seemed most important, and the inner and outer setting played a role as well. Furthermore, determinants interacted in explaining outcomes. For example, we found that attitude and role identity interacted in explaining intentions.

Role identity emerged as an important variable, given that participants with stronger role identities had stronger intentions to use the guideline and provided more quit advice. It is imperative that all groups of HCPs come to perceive Ask-Advise-Refer as their task, such that every smoker visiting a HCPs will be advised to quit smoking and referred to adequate care [3, 4]. The limited (time) investment required for Ask-Advise-Refer may help HCPs to perceive SCC as fitting with their profession. Furthermore, role identity might be strengthened when HCPs come to perceive smoking as a disease (which they typically treat) rather than a habit (which they may leave to the patient). In line with this, literature on shared responsibility bias shows that the so-called bystander effect (i.e., reduced sense of responsibility when others are present who could take responsibility) is less pronounced when the situation is perceived as more dangerous [19].

The results point to other routes to improving SCC as well. It is important that efforts are targeted at HCP group, given that barriers encountered, intentions to use the guideline, and implementation of the Ask-Advise-Refer tasks differed between groups. Furthermore, many participants in this study identified lack of training as a barrier to providing SCC. However, participation in training has its own barriers, including lack of time or interest, and other priorities [61]. Given time constraints and low levels of role identity found in this study, it is important that training is attractive, relevant (e.g., addressing barriers important for the specific context) and preferably time efficient [62]. The current results also suggest that training should focus on skills (which was positively associated with intentions to use the guideline and advising to quit), rather than knowledge (which was not significantly associated with any outcome). It is likely that many HCPs already know the disadvantageous effects of smoking and are generally aware of interventions that may help smokers quit, but perceive themselves to lack the skills required to implement SCC, for example with regard to motivating patients or addressing sensitive subjects. Furthermore, organizational changes may also facilitate SCC, in particular increasing familiarity with the guideline and arranging collaborations for SCC with primary care [63]. Such collaborations were associated with more quit advice, and doubled referral rates among groups of HCPs that were overall less likely to refer. However, given the cross-sectional nature of this study, it is also possible that those who adequately implement SCC familiarized themselves with relevant guidelines and arranged collaborations, rather than the other way around.

This study has limitations. First, there might be some selection bias in our sample, as HCPs who are interested in and motivated to provide SCC might have been more willing to participate in our study. We have attempted to mitigate this risk by inviting HCPs regardless of their experience with SCC, recruiting participants in many ways including through their professional associations and colleagues, and ensuring anonymity and confidentiality. In order to ensure representative subgroups of HCPs, we had to focus our recruitment strategy on a number of HCP groups that were considered most relevant for the current study. Future studies among other types of HCPs (e.g. nurses, psychologists) are recommended. Second, as is common in this type of research, results were based on self-report, which may have resulted in socially desirable answers. Although other methods such as observation would reduce social desirability bias, they would also have reduced our sample size considerably. Third, the cross-sectional nature of this study did not allow for causal interpretations. Fourth, although most domains of the CFIR were covered in this study, we did not assess the process of implementation (e.g., whether participants were involved in the adoption process). However, factors that previous studies have shown to be important for the implementation of SCC were included in this study, and the implementation process did not emerge in participants’ responses to the open-ended question about barriers to implementation suggesting that other factors are more important. Finally, only Dutch HCPs were included, but correspondence of our findings with the international literature, and strong similarities between the Dutch Tobacco Dependence Guideline and international guideline [12], suggest that findings are generalizable to other high-income countries.

Notwithstanding these limitations, this study has important implications. There is much to be gained in SCC, given that implementation of Ask-Advise-Refer is still relatively low. In order to ensure that every smoker is advised to quit smoking and offered adequate care changes are needed at the level of the HCP (i.e., facilitate role identity and skills) and the organization (i.e., facilitate collaboration agreements and guideline familiarity). Future implementation strategies should be targeted to the specific barriers encountered by HCPs, the contexts that they work in, and the patients that they work with. Strategy development could be informed by the Behaviour Change Wheel and its taxonomy, which provides an evidence-based method to the use of behaviour change techniques [64].

## Conclusions

Although smoking cessation guidelines are widely available, implementation of smoking cessation in practice, and in specific the implementation of Ask-Advise-Refer, remains relatively low. To improve the provision of smoking cessation care, several barriers need to be addressed at different system levels. Implementation strategies should aim to improve the smoking cessation related role identity and skills of the healthcare provider, should aim to improve guideline accessibility and familiarity and facilitate organization-level commitment for and formal ratification of smoking cessation care.

## Supplementary information

**Additional file 1:** **Table S1.** Recruitment strategy per profession (*N* = 883). **Table S2.** Scores on psychosocial variables by profession (*N* = 883). **Table S3A.** Means and standard deviations of responders and drop-outs on background and outcome variables, accompanied by *t*-statistics testing differences between groups. **Table S3B.** Frequencies and percentages of responders and drop-outs on background variables, accompanied by *χ*^2^-statistics testing differences between groups. **Table S4.** Dosage delivered of other smoking cessation counselling tasks (*N* = 883).

## Data Availability

The datasets used and/or analyzed during the current study are available from the corresponding author on reasonable request.

## Abbreviations

CCR: Correct classification rate
COPD: Chronic obstructive pulmonary disease
GP: General practitioner
HCP: Healthcare provider
RQ: Research question
SCC: Smoking cessation care
