# Supplementary material for: Facilitating smoking cessation in patients who smoke: a large-scale cross-sectional comparison of fourteen groups of healthcare providers
Source: BMC Health Serv Res. 2019 Oct 25;19:750. doi: 10.1186/s12913-019-4527-x (PMC6815021; doi:10.1186/s12913-019-4527-x)
Supplement: Supplementary file 1 — Additional file 1: Table S1. Recruitment strategy per profession (N = 883). Table S2. Scores on psychosocial variables by profession (N = 883). Table S3A. Means and standard deviations of responders and drop-outs on background and outcome variables, accompanied by t-statistics testing differences between groups. Table S3B. Frequencies and percentages of responders and drop-outs on background variables, accompanied by χ2-statistics testing differences between groups. Table S4. Dosage delivered of other smoking cessation counselling tasks (N = 883). [file 12913_2019_4527_MOESM1_ESM.docx]

Table 1. Recruitment strategy per profession (*N* = 883).

|  |  | **N** | Professional association | Colleague | Direct e-mail to department/practice | Dutch Medical Journal (NTvG) | Academic primary care netwrok | Royal Dutch Medical Association | Social media | Physicians popular magazine (Arts en Auto) | Other | Unknown |
| --- | --- | --- | --- | --- | --- | --- | --- | --- | --- | --- | --- | --- |
| *Physicians* | |  |  |  |  |  |  |  |  |  |  |  |
|  | Addiction specialist^a^ | 45 | **24** | 9 | 1 | 1 |  |  |  | 1 | 1 | 8 |
|  | Anaesthesiologist | 62 | 13 | **33** | 5 | 3 |  | 3 | 1 | 1 |  | 3 |
|  | Cardiologist | 52 | 1 | 20 | **24** |  |  | 3 | 1 |  |  | 3 |
|  | GP | 148 | **43** | 25 |  | 18 | 33 | 7 | 3 | 6 | 6 | 7 |
|  | Internist | 63 | **26** | 21 | 8 | 4 |  | 1 |  |  | 1 | 2 |
|  | Neurologist | 63 | 6 | **23** | **23** | 2 |  | 3 |  |  |  | 6 |
|  | Paediatrician^a^ | 36 | **16** | 8 | 6 | 3 |  | 3 |  |  |  |  |
|  | Pulmonologist | 102 | **76** | 11 | 11 | 1 |  | 2 |  |  |  | 1 |
|  | Other | 31 | 1 |  |  |  |  |  | 4 |  |  | **26** |
|  | Ophthalmologist | 16 | **14** | 2 |  |  |  |  |  |  |  |  |
|  | Surgeon | 68 | 11 | **40** | 4 | 5 |  | 2 | 3 |  |  | 3 |
|  | Youth specialist | 48 | **25** | 13 | 3 | 3 |  | 1 | 1 | 1 | 1 |  |
| *Other healthcare professionals* | |  |  |  |  |  |  |  |  |  |  |  |
|  | Dental hygienist^b^ | 58 | **58** |  |  |  |  |  |  |  |  |  |
|  | Dentist^b^ | 26 | **26** |  |  |  |  |  |  |  |  |  |
|  | Midwife | 65 | **58** | 5 |  |  |  |  |  | 1 | 1 |  |
| **Total** | | 883 | 398 | 210 | 85 | 40 | 33 | 25 | 13 | 10 | 10 | 59 |
| *Note.* The main recruitment strategy per profession is indicated in bold.  a. Participants were recruited through an e-mail sent by the association and at a conference/symposium organized by the association.  b. Representative samples of 400 dental hygienists and dentists were drawn and approached by the respective professional associations. | | | | | | | | | | | | |

Table 2. Scores on psychosocial variables by profession (*N* = 883).

|  |  |  | *M (SD)* | | | | | | | |
| --- | --- | --- | --- | --- | --- | --- | --- | --- | --- | --- |
|  |  | n | Attitude | Knowledge | Skills | Social support | Role identity | Outcome expectations | Agreement content |  |
| *Physicians* | |  |  |  |  |  |  |  |  |  |
|  | Addiction specialist | 45 | 3.93 (0.81) | 3.87 (0.97) | 3.98 (0.85) | 3.33 (1.09) | **4.42 (0.81)** | 3.84 (0.80) | 3.73 (0.75) |  |
|  | Anaesthesiologist | 62 | 3.65 (0.83) | 2.40 (1.15) | 2.97 (1.01) | 2.74 (0.92) | 2.81 (1.01) | 3.05 (0.86) | 3.16 (0.41) |  |
|  | Cardiologist | 52 | 3.60 (0.85) | 2.69 (1.00) | 3.19 (0.91) | 2.79 (0.78) | 3.31 (0.85) | 3.31 (0.61) | 3.17 (0.47) |  |
|  | GP | 148 | 3.73 (0.82) | 3.45 (0.92) | 3.57 (0.87) | 3.21 (0.94) | 3.68 (1.03) | 3.45 (0.85) | 3.76 (0.68) |  |
|  | Internist | 63 | 3.38 (0.75) | 2.54 (0.93) | 3.13 (0.83) | 2.63 (0.77) | 3.43 (1.03) | 3.25 (0.72) | 3.10 (0.50) |  |
|  | Neurologist | 63 | 3.56 (0.76) | 2.33 (0.90) | 2.87 (0.66) | 2.70 (0.75) | 3.00 (0.90) | 3.19 (0.59) | 3.05 (0.21) |  |
|  | Paediatrician | 36 | 3.89 (0.75) | 2.64 (0.99) | 2.89 (0.95) | 3.11 (1.06) | 3.75 (0.94) | 3.31 (0.79) | 3.47 (0.65) |  |
|  | Pulmonologist | 102 | 3.58 (0.80) | 3.09 (1.08) | 3.40 (0.84) | 3.01 (1.03) | 3.77 (0.91) | 3.29 (0.80) | 3.37 (0.69) |  |
|  | Other | 31 | 3.65 (1.02) | 2.84 (1.13) | 3.16 (1.10) | 2.68 (1.01) | 3.42 (1.23) | 3.35 (0.61) | 3.13 (0.67) |  |
|  | Ophthalmologist | 16 | 3.31 (0.95) | 2.13 (0.96) | 2.81 (0.66) | 2.69 (1.01) | 2.94 (0.93) | 3.25 (0.68) | 3.13 (0.67) |  |
|  | Surgeon | 68 | 3.44 (0.66) | 2.41 (0.93) | 3.00 (0.86) | 2.68 (0.76) | 3.09 (0.88) | 3.21 (0.64) | 3.13 (0.42) |  |
|  | Youth specialist | 48 | 3.90 (0.86) | 2.60 (1.01) | 2.90 (0.81) | 2.65 (0.70) | 3.67 (0.86) | 3.42 (0.74) | 3.23 (0.56) |  |
| *Other healthcare professionals* | |  |  |  |  |  |  |  |  |  |
|  | Dental hygienist | 58 | 3.62 (0.88) | 2.67 (1.03) | 3.17 (1.08) | 2.95 (0.76) | 3.67 (0.87) | 3.29 (0.65) | 3.33 (0.54) |  |
|  | Dentist | 26 | 3.50 (0.58) | 2.38 (1.27) | 2.81 (1.10) | 2.92 (0.63) | 3.54 (0.65) | 3.12 (0.77) | 3.19 (0.49) |  |
|  | Midwife | 65 | **4.70 (0.85)** | 3.62 (1.03) | 3.52 (0.97) | 3.12 (0.91) | **4.06 (1.01)** | 3.35 (1.05) | 3.20 (0.47) |  |
| ***Total*** | | 883 | 3.71 (0.86) | 2.89 (1.11) | 3.24 (0.94) | 2.92 (0.91) | 3.54 (1.02) | 3.32 (0.78) | 3.33 (0.61) |  |
| *Note.* Mean scores ≥ 4 (i.e., ‘agree’ or ‘strongly agree’) are indicated in bold. | | | | | | | | | | |

Table 3A. Means and standard deviations of responders and drop-outs on background and outcome variables, accompanied by *t*-statistics testing differences between groups.

|  | ***M (SD)*** | |  |
| --- | --- | --- | --- |
| **Variables** | **Responders**  **(*n* = 883)** | **Drop-outs**  **(*n* = 280-337)** | ***t*-statistics** |
| Age | 45.72 (10.84) | 45.85 (12.74) | *t*(1216) = 0.18, *p* = .86, *d* = 0.01 |
| Years worked | 13.24 (10.25) | 14.11 (10.97) | *t*(1160) = 1.22, *p* = .22, *d* = 0.08 |
| Intention to use guideline | 3.50 (0.03) | 3.32 (0.72) | *t*(1001) = 2.26, *p* = .02, *d* = 0.25 |
| *Note.* For each variable attrition analyses were performed on all participants for whom data on this particular variable was available. | | | |

Table 3B. Frequencies and percentages of responders and drop-outs on background variables, accompanied by *χ*^2^-statistics testing differences between groups.

|  |  | ***n* (%)** | |  |
| --- | --- | --- | --- | --- |
| **Variable** | **Categories** | **Responders** | **Drop-outs** | ***χ*²-statistic** |
| Profession | Addiction specialist | 45 (74%) | 16 (26%) | *χ*²(14) = 95.33, *p* < .001, *V* = .28 |
|  | Anaesthesiologist | 62 (85%) | 11 (15%) |  |
|  | Cardiologist | 52 (83%) | 11 (18%) |  |
|  | GP | 148 (83%) | 31 (17%) |  |
|  | Internist | 63 (77%) | 19 (23%) |  |
|  | Neurologist | 63 (82%) | 14 (18%) |  |
|  | Paediatrician | 36 (71%) | 15 (29%) |  |
|  | Pulmonologist | 102 (75%) | 35 (26%) |  |
|  | Other physician | 31 (43%)*** | 42 (58%)*** |  |
|  | Ophthalmologist | 16 (62%) | 10 (39%) |  |
|  | Surgeon | 68 (83%) | 14 (17%) |  |
|  | Youth specialist | 48 (60%) | 32 (40%)* |  |
|  | Dental hygienist | 58 (53%)** | 52 (47%)*** |  |
|  | Dentist | 26 (58%) | 19 (42%) |  |
|  | Midwife | 65 (78%) | 18 (22%) |  |
| Gender | Male | 372 (76%) | 118 (24%) | *χ*²(1) = 5.14, *p* = .02, *V* = .07 |
|  | Female | 511 (70%) | 219 (30%) |  |
| Dosage ask (all) | No | 383 (43%) | 65 (38%) | *χ*²(1) = 1.54, *p* = .22, *V* = .04 |
|  | Yes | 500 (57%) | 105 (62%) |  |
| Dosage advise (all/majority) | No | 508 (58%) | 102 (60%) | *χ*²(1) = 0.36, *p* = .55, *V* = .02 |
|  | Yes | 375 (43%) | 68 (40%) |  |
| Dosage refer (all/majority) | No | 450 (51%) | 87 (58%) | *χ*²(1) = 2.29, *p* = .13, *V* = .05 |
|  | Yes | 433 (49%) | 64 (42%) |  |
| *Note.* For each variable attrition analyses were performed on all participants for whom data on this particular variable was available.  * Deviation from the expected cell count at *p* < .05.  ** Deviation from the expected cell count at *p* < .01.  *** Deviation from the expected cell count at *p* < .001. | | | | |

Table 4. Dosage delivered of other smoking cessation counselling tasks (*N* = 883).

|  |  |  | **All** | **Smokers** | | | **Smokers motivated to quit** | | | | |  | **Advise to quit** | | | |
| --- | --- | --- | --- | --- | --- | --- | --- | --- | --- | --- | --- | --- | --- | --- | --- | --- |
|  |  | **n** | Register smoking status | Take smoking profile | Assess quit motivation | Register quit motivation | Discuss barriers | Make a quit plan | Advise/prescribe pharmacotherapy | Arrange follow-up | Short motivational intervention* | | New patients | Smoking-related complaints | Pre-surgery | Pregnant |
| *Physicians* | |  |  |  |  |  |  |  |  |  |  | |  |  |  |  |
|  | Addiction specialist | 45 | **78%** | **56%** | **89%** | **78%** | **82%** | **71%** | **78%** | **80%** | **78%** | | 44% | **69%** | **54%** | **85%** |
|  | Anaesthesiologist | 62 | **73%** | 44% | 11% | 2% | 2% | 0% | 3% | 0% | 7% | | 15% | 32% | 28% | **55%** |
|  | Cardiologist | 52 | **67%** | 40% | **69%** | 37% | 48% | 23% | 4% | 27% | 37% | | **71%** | **82%** | **53%** | **94%** |
|  | GP | 148 | 14% | 12% | **65%** | **52%** | **77%** | **55%** | **57%** | **69%** | **76%** | | 20% | **72%** | 14% | **90%** |
|  | Internist | 63 | **84%** | **56%** | **57%** | 44% | 48% | 21% | 10% | 22% | 29% | | 46% | **78%** | 41% | **96%** |
|  | Neurologist | 63 | **52%** | 27% | 32% | 19% | 24% | 8% | 6% | 19% | 18% | | 22% | **76%** | 46% | **79%** |
|  | Paediatrician | 36 | 19% | 17% | 33% | 42% | 42% | 19% | 3% | 33% | 28% | | 36% | **55%** | 27% | **65%** |
|  | Pulmonologist | 102 | **90%** | **78%** | **83%** | **66%** | **60%** | 24% | 27% | **53%** | **54%** | | **77%** | **84%** | **59%** | **94%** |
|  | Other | 31 | 42% | 29% | 39% | 32% | 42% | 26% | 23% | 29% | 29% | | 35% | **70%** | 35% | **90%** |
|  | Ophthalmologist | 16 | 0% | 0% | 13% | 19% | 6% | 0% | 0% | 6% | 13% | | 13% | **67%** | 0% | **50%** |
|  | Surgeon | 68 | 49% | 37% | 47% | 30% | 24% | 7% | 7% | 18% | 41% | | 48% | **65%** | **58%** | **88%** |
|  | Youth specialist | 48 | 17% | 15% | 38% | 29% | 35% | 8% | 2% | 10% | 21% | | 35% | **64%** | 14% | 44% |
| *Other healthcare professionals* | |  |  |  |  |  |  |  |  |  |  | |  |  |  |  |
|  | Dental hygienist | 58 | **66%** | 36% | 45% | 47% | 53% | 14% | 10% | 12% | 41% | | 50% | **64%** | **72%** | **76%** |
|  | Dentist | 26 | **62%** | 35% | 46% | 31% | 39% | 12% | 8% | 19% | 27% | | 39% | **61%** | **61%** | **81%** |
|  | Midwife | 65 | **99%** | **86%** | **91%** | **86%** | **77%** | **57%** | 6% | **62%** | **74%** | |  |  |  |  |
| *Note.* Percentages refer to participants who performed the task among ‘all’ relevant patients (for registering smoking status and taking smoking profile, and advising specific groups of smokers to quit), or among ‘all or the majority’ of relevant patients (for the other tasks). GP = general practitioner; SCC = smoking cessation counselling. *** Providing a brief motivational interventions was asked concerning smokers who consider quitting. Percentages ≥ 50 are indicated in bold. Percentages are valid percentages; participants could indicate ‘not applicable’ for providing quit advice to specific groups (not assessed among midwives). | | | | | | | | | | | | | | | | |
